# Synthesis and evaluation of new amidrazone-derived hydrazides as a potential anti-inflammatory agents

**DOI:** 10.1007/s00706-018-2197-8

**Published:** 2018-06-27

**Authors:** Renata Paprocka, Małgorzata Wiese-Szadkowska, Anna Helmin-Basa, Liliana Mazur, Jolanta Kutkowska, Jacek Michałkiewicz, Bożena Modzelewska-Banachiewicz, Leszek Pazderski

**Affiliations:** 1 Department of Organic Chemistry, Faculty of Pharmacy, Nicolaus Copernicus University in Toruń, Bydgoszcz, Poland; 2 Department of Immunology, Faculty of Pharmacy, Nicolaus Copernicus University in Toruń, Bydgoszcz, Poland; 3 Faculty of Chemistry, Maria Curie-Skłodowska University, Lublin, Poland; 4 Department of Genetics and Microbiology, Maria Curie-Sklodowska University, Lublin, Poland; 5 Department of Clinical Microbiology and Immunology, The Children’s Memorial Health Institute, Warsaw, Poland; 6 Department of Analytical Chemistry and Applied Spectroscopy, Faculty of Chemistry, Nicolaus Copernicus University in Toruń, Toruń, Poland

**Keywords:** Drug research, Anti-inflammatory activity, Antiproliferative agents, Acylation, Crystal structure

## Abstract

**Abstract:**

The series of new hydrazide derivatives were synthesized in reactions of N^3^-substituted amidrazones with cyclic anhydrides as potential anti-inflammatory and antibacterial agents. The compounds were characterized by ^1^H-^13^C two-dimensional NMR techniques, which revealed the presence of two tautomeric forms in DMSO-*d*_6_ solutions, while the molecular structure of one species was confirmed by single-crystal X-ray diffraction. The anti-inflammatory effects of hydrazides on peripheral blood mononuclear cells were experimentally evaluated. Three compounds showed antiproliferative activity comparable to ibuprofen. One derivative demonstrated strong reduction of lymphocyte proliferation stimulated by anti-CD3 antibody (by 90%) and PHA, as well as low cell toxicity. The obtained compounds exhibited relatively weak antibacterial activity; they were more effective against Gram-positive bacterial strains.

**Graphical abstract:**

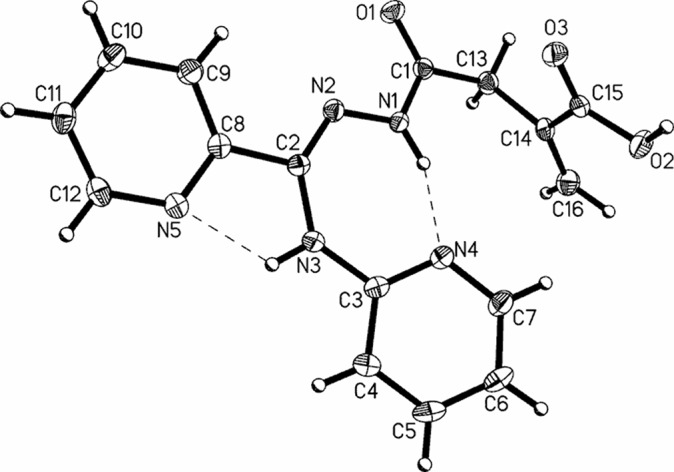

**Electronic supplementary material:**

The online version of this article (10.1007/s00706-018-2197-8) contains supplementary material, which is available to authorized users.

## Introduction

Non-steroidal anti-inflammatory drugs (NSAID) belong to the most popular therapeutic agents [1]. A classical example of NSAID is ibuprofen used in many medical conditions from headache, rheumatoid arthritis, cephalgia to muscular strain [2]. Moderate antimicrobial activity of ibuprofen has also been reported [3]. Like all profen drugs, ibuprofen possesses the chiral carbon atom within the propionic acid moiety. The majority of sold ibuprofen drugs are racemic mixtures, although only S enantiomer (dexibuprofen) is associated with anti-inflammatory effects [4]. However, cardiovascular and gastrointestinal risks suggest more caution in the common use of ibuprofen and other NSAIDs even available without prescription [5].

Amidrazone derivatives are known for their wide biological effects: bacteriostatic, antiviral, antiproliferative, antitumor, anti-inflammatory, antinociceptive, and anticonvulsant [6–11]. They are used in the synthesis of many heterocyclic compounds [12]. In our recent studies, we reported amidrazone derivatives possessing methacrylic acid moiety: 1,2,4-triazole derivatives with anti-inflammatory activity comparable to ibuprofen [13] as well as hydrazides inhibiting the production of proinflammatory cytokine TNF-α [14]. On the other hand, hydrazide derivatives [15–17] and drugs possessing hydrazide moiety (nitrofural, nifuroxazide, isoniazid) demonstrated essential antimicrobial activity.

Continuing our study on N^3^-substituted amidrazones, we focused on the synthesis of hydrazides possessing achiral methacrylic acid moiety similar to propionic acid present in ibuprofen. Taking into account the side effects of common NSAID drugs and the growing number of bacterial strains resistant to available antibiotics [18, 19], searching for new potential drugs still constitutes an actual task. The aim of this work was to synthesize new potentially active compounds and estimate their anti-inflammatory effects on peripheral blood mononuclear cells (PBMC) as well as their antibacterial properties.

## Results and discussion

### Formation and general characterization of **5**–**8**

The series of new hydrazides **5**–**8** was obtained in the reaction of N^3^-substituted amidrazones **1**–**4** [20] with itaconic anhydride, carried out in anhydrous diethyl ether (Scheme 1).

**Figure:**
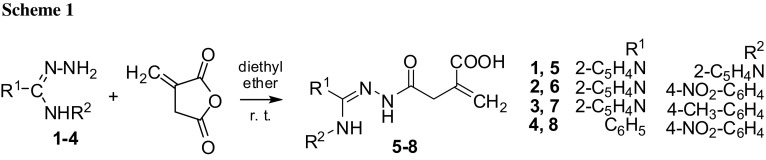

Isolation of acyclic compounds **5**–**8** was possible only at a short time of reaction, i.e., 2 h (instead of 7 days which resulted in the formation of the previously described 1,2,4-triazole derivatives [13]). The purity and correctness of their empiric formulae was checked by elemental analyses, which exhibited that **6** appeared in the dihydrate form.

The molecular structures of **5**–**8** were confirmed by IR in the solid phase as well as by ^1^H and ^13^C NMR in DMSO-*d*_6_ (which involved also ^13^C DEPT and two-dimensional ^1^H-^13^C HMQC and HMBC measurements, allowing the attribution of all proton and carbon resonances). However, in DMSO-*d*_6_ solutions, the equilibrium of two tautomeric forms: amide-hydrazone (**A**) and hydrazide imide (**B**) [21], having partly different *δ*^1^H and *δ*^13^C chemical shifts, was observed for all **5**–**8** compounds (Scheme 2).

**Figure:**
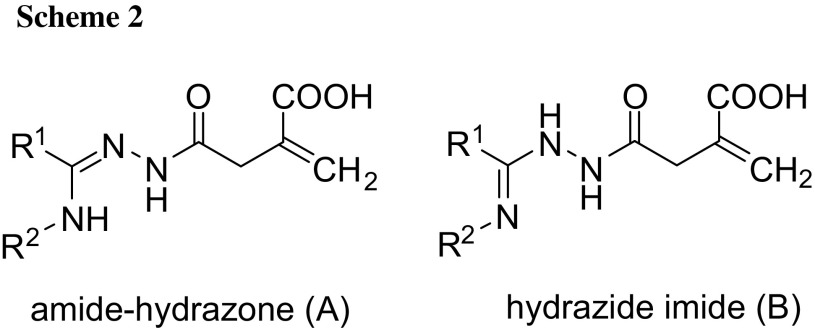

## ^1^H and ^13^C NMR spectroscopy of **5**–**8**

The analysis of ^1^H, ^13^C, ^13^C DEPT, ^1^H-^13^C HMQC, and ^1^H-^13^C HMBC-NMR spectra reveals that **5**–**8** appear in DMSO-*d*_6_ solutions as mixtures of two tautomeric forms, most-likely amide-hydrazone (**A**, Scheme 2, left) and hydrazide imide (**B**, Scheme 2, right); at 300 K, these **A** and **B** species remain at equilibrium with nearly the same ratio of 55:45%, for all **5**–**8** compounds (as determined by integration of the best separated ^1^H resonances, preferably those of –NHCO–). This tautomerism generally results in the appearance of different ^1^H and ^13^C aliphatic signals for **A** and **B**, whereas the aromatic ^1^H and ^13^C ones (deriving from R^1^ and R^2^ rings) remain identical. The separation of ^1^H resonances is especially well observed for the –NHCO– hydrogens, while the –NH– peaks are either well separated (**6**–**8**) or shared (**5**) by **A** and **B**, probably depending on the rate of **A** ↔ **B** conversion (i.e., the rate of the proton transfer between the respective nitrogen), with respect to the applied NMR timescale.

The *δ*^1^H parameters confirm the proposed molecular structures of **5**–**8**. In particular, the broad ca. 12.5 ppm peaks, as well as more narrow ca. 9.75–11.4 ppm and ca. 8.45–9.4 ppm singlets correspond well to the H atoms in the –COOH, –NH–CO–, and –NH– groups, respectively, whereas ca. 5.6–6.2 ppm range is typical for vinyl = CH_2_ hydrogens. The methylene –CH_2_– protons have relatively high ca. 3.25–3.75 ppm values, reflecting the adjacency of the >C=O and =CH_2_ groups (both yield inductive and/or anisotropic deshielding effects). Finally, the signals within ca. 6.65–8.5 ppm range are characteristic for various CH atoms present in the studied phenyl, 4-methylphenyl, 4-nitrophenyl, and 2-pyridyl aromatic rings.

Also, the *δ*^13^C parameters are consistent with the assumed molecular structures of **5**–**8**. The appearance of –COOH and –NH–CO– signals in ca. 166–173 ppm range is typical for carboxylic and carbonamide carbons. The high values of ca. 136–137 ppm, ca. 139–143 ppm, and ca. 127–128 ppm, observed for both types of >C= atoms and those of =CH_2_, respectively, reflect unsaturated properties of these aliphatic carbons. In contrast, much lower parameters for the methylene –CH_2_– carbon, being ca. 28–38 ppm, are caused by its saturated character. Finally, the signals within ca. 115–155 ppm range are characteristic for aromatic carbons, these chemical shifts being generally larger for the substituted C atoms than for the CH ones.

### Crystal and molecular structure of **5**

**Fig. 1:**
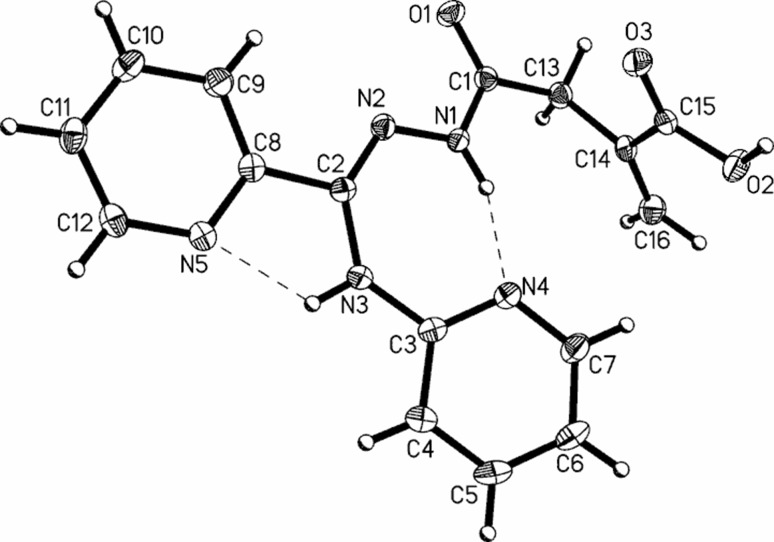
A perspective view of **5** showing the atom-numbering scheme. Displacement ellipsoids are drawn at the 50% probability level. Dashed lines indicate the hydrogen bonds

The molecular structure of **5** (which can be treated as a model system for all **5**–**8** compounds) was studied by single-crystal X-ray diffraction. The data reveal that **5** crystallizes in the centrosymmetric space group *P*2_1_/c with one molecule in the asymmetric part of the unit cell (Fig. 1). The relevant geometric parameters (Table S4, Supplementary Material) indicate that in the solid phase **5** appears in the amide-hydrazone tautomeric form, in which the molecules adopt the *Z*-*anti* configuration around the imine C2=N2 and amide C1–N1 bonds, respectively.

The central acylamidrazone (O1>>N3) unit is almost planar, with small rotation around the azine N1–N2 bond and is almost co-planar with C2-substituted 2-pyridyl ring. In turn, the 2-aminopyridine moiety is slightly twisted out of the plane of the spacer unit as confirmed by the N2–C2–N3–C3 torsion angle, being 18.7(2)°. The distortion can be explained by steric hindrance between the pyridyl ring and the methacrylic acid unit. The carboxyl group is twisted by 8.1(1)° from the plane of C13/C14/C16 atoms and forms a dihedral angle of 78.7(1)° with the best plane of the hydrazide moiety.

**Fig. 2:**
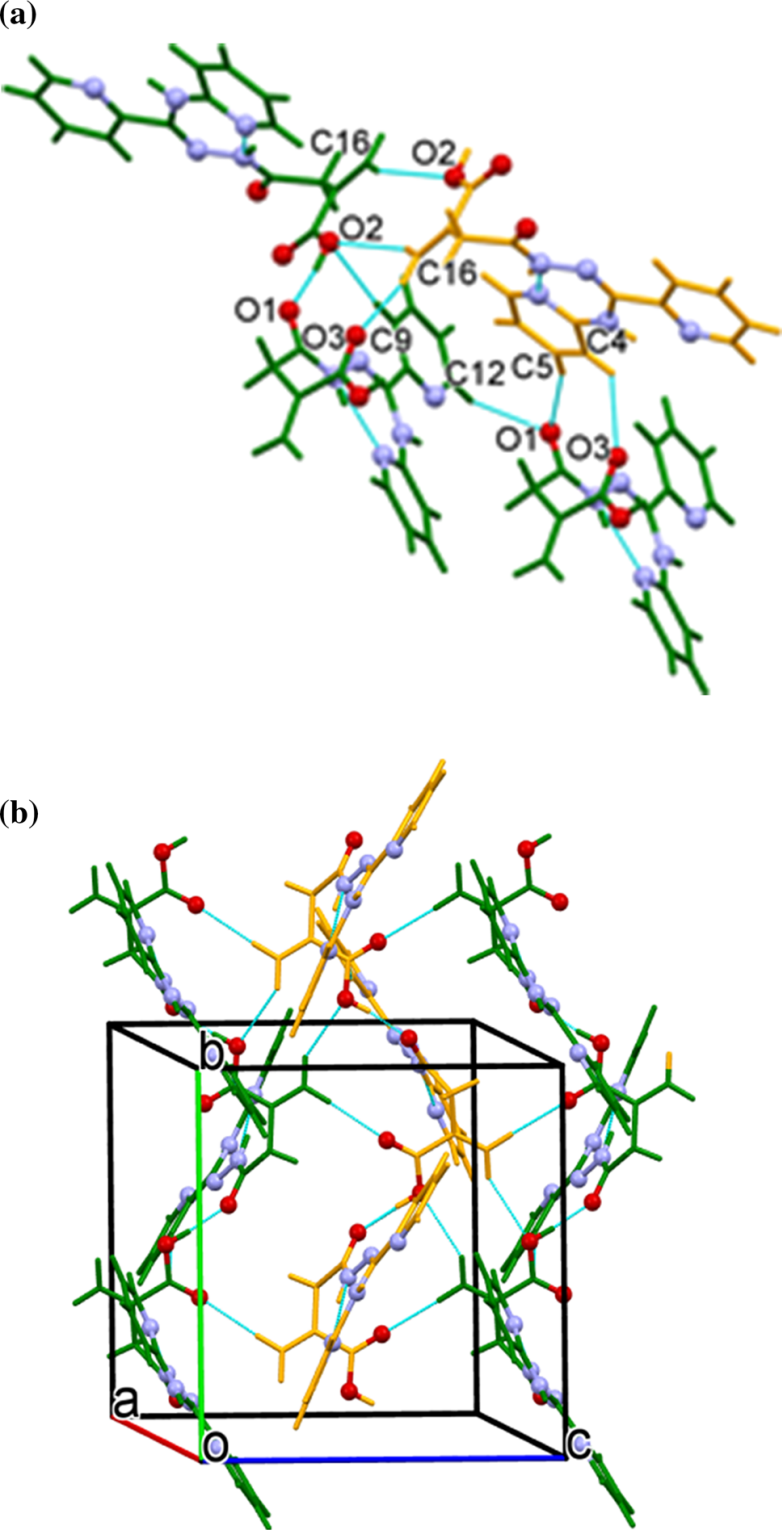
Part of the crystal structure of **5** showing: **a** intermolecular interaction patterns; **b** hydrogen-bonded helical chains linked via C–H…O contacts into the (100) molecular layer

The primary supramolecular motifs in crystal **5** are molecular chains (Fig. 2b) generated by 2_1_ screw axis-related molecules, linked by the strong O2–H2…O1 (2.594(2) Å, 168(1)°) hydrogen bonds. The relative orientation of the adjacent inversion-related chains enables creation of quite short, linear C16–H16a…O3 and C16–H16b…O2 hydrogen bonds (Table S5, Supplementary Material). The resulting (100) molecular layers are stabilized by aryl–carboxyl and aryl–aryl C–H…O/π contacts (Fig. 2a) leading to the complex 3D supramolecular architecture.

### Anti-inflammatory activity of **5**–**8**

The influence of compounds **5**–**8** at concentrations 1, 10, and 50 µg/cm^3^ on the viability of PBMC was evaluated. Compounds **5** and **7** showed low toxicity (Fig. S1, Supplementary Material). Derivatives **6** and **8** possessing the nitro group induced stronger cell apoptosis at the highest concentration 50 µg/cm^3^ (more than 30% of cells in apoptosis).

**Fig. 3:**
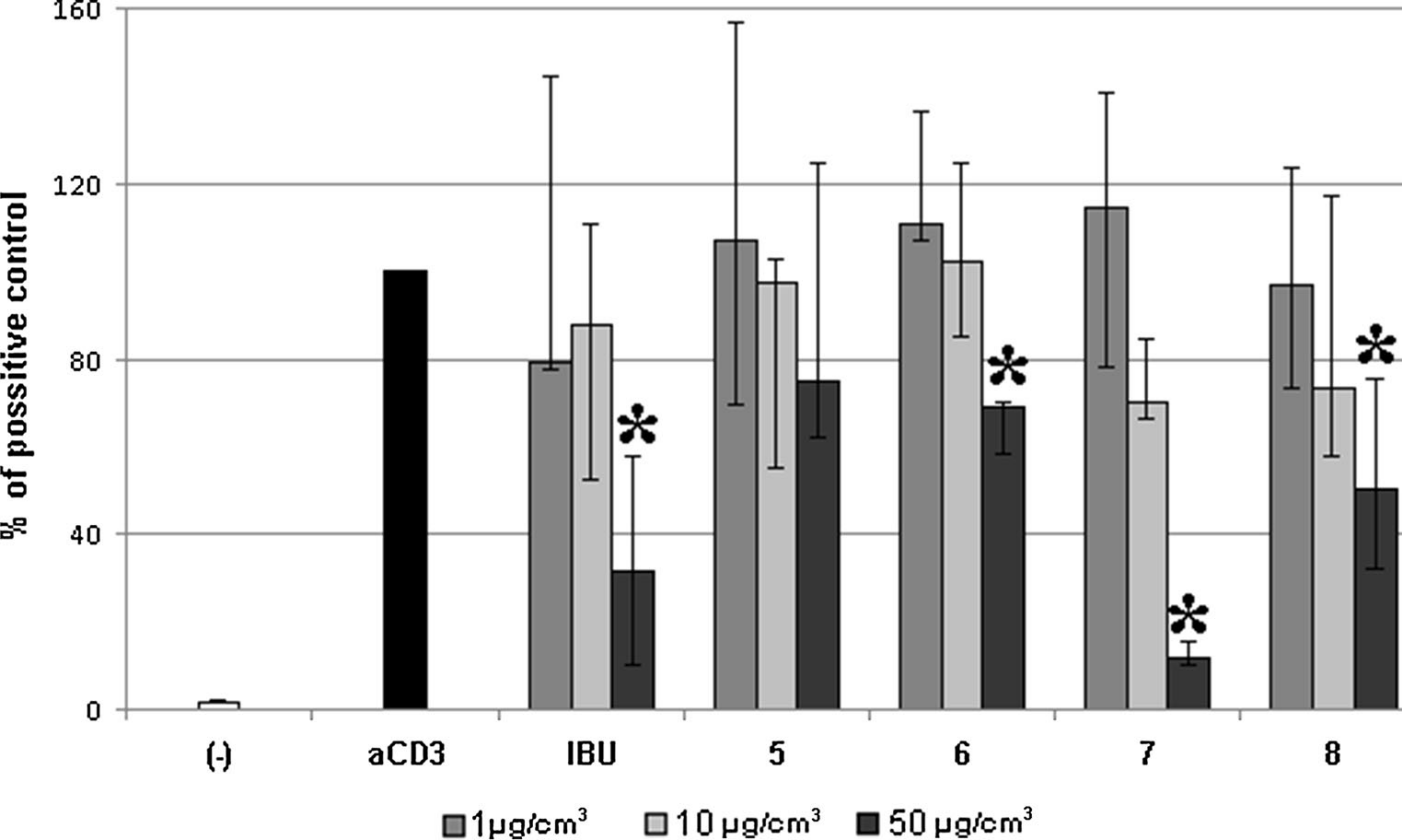
The influence of compounds **5**–**8** on the proliferation of human peripheral blood mononuclear cells (PBMC) induced by the anti-CD3 antibody. Cells were treated with anti-CD3 antibody (4 μg/cm^3^) and compounds **5**–**8** at concentrations 1, 10, and 50 μg/cm^3^. Ibuprofen (IBU) was used as reference drug; negative control (−)—non-stimulated PBMC. After 72 h of incubation, the proliferation of PBMC was measured using ^3^H thymidine incorporation assay. The results are shown as percentage of positive control (anti-CD3 antibody-stimulated PBMC). Values are expressed as medians from five independent experiments and interquartile ranges (Q1–Q3). Asterisk indicates significant differences compared to positive control at *p* < 0.05; hash indicates significant difference compared to IBU at *p* < 0.05 (*n* = 4–6)

Compounds **5**–**8** showed no significant influence on the proliferation of non-stimulated PBMC. However, three derivatives: **6**–**8** significantly inhibited the proliferation of mouse monoclonal anti-CD3 antibody-stimulated PBMC comparable to ibuprofen (but only at concentration 50 µg/cm^3^). The strongest inhibitor was **7** possessing 2-pyridine and methylphenyl substituents (inhibition about 90%; Fig. 3).

Polyclonal lymphocyte activators induce mitotic proliferation in PBMC. The measurements of proliferation and cytokine production in response to mitogens and specific antigens help to understand the mechanism of immune response. Anti-CD3 antibodies are very potent mitogens of T lymphocytes; they induce their proliferation, production, and secretion of some cytokines such as TNF-α, INF-α, and IL-10. Anti-CD3 antibodies induced T-lymphocyte activation is associated with signaling pathway, including CD3, ZAP70-phospholipase C-*γ*1 and mitogen-activated protein kinase/c-Jun N-terminal kinase [22, 23]. Hence, the low responsiveness of T lymphocyte to CD3 antibodies indicated that derivatives **6**–**8** can block this polyclonal lymphocyte activation. Furthermore, it suggests that these hydrazides modulate the synthesis of some cytokines and the signaling pathway that we mentioned above.

Additionally, the effect of compounds **5**–**8** on PBMC proliferation stimulated by polyclonal stimulus phytohaemagglutinin (PHA, lectins of most T lymphocytes) was tested. Compound **6** at concentration 50 µg/cm^3^ inhibited PBMC proliferation by about 30%. The strongest suppression was once more demonstrated by compound **7** at concentration 50 µg/cm^3^ (99% inhibition; Fig. S2, Supplementary Material). PHA binds to cell membrane and activates adenylate cyclase or guanylate-cyclase, which transduce signal from the membrane to the nucleus of lymphocytes [24]. These results suggest that T lymphocytes might be also influenced by derivatives **6** and **7** when using PHA, a different T cell mitogen. These observations point to that compound **7** affected on two different lymphocyte activation pathways (PHA and CD3).

To examine in detail the properties of the most promising anti-inflammatory compound **7,** we used the Apoptosis, DNA Damage and Cell Proliferation Kit (BD Pharmingen™). This test gave the opportunity to examine the viability of the cells [expression of cleaved fragment of PARP—poly (ADP-ribose) polymerase—a marker of cellular apoptosis] and synthesis of DNA by the expression of: (a) BrdU—an analog of the DNA precursor thymidine (check the proliferation statue); (b) γH2AX—histone H2AX phosphorylated on Ser 139—that detects double-stranded DNA breaks; (c) total DNA for cell cycle analysis (staining with DAPI solution).

A low percentage of PARP-positive cells confirmed the lack of toxicity in cells cultured with compound **7** alone or together with PHA (Fig. S3, Supplementary Material). Further cytometric analyses revealed that PBMCs cultured with compound **7** (regardless of doses) and PHA had shown: (a) decreasing percentage of BrdU-incorporated cells (Fig. S4, Supplementary Material); (b) reduced percentage of cells with γH2AX expression (Fig. S5, Supplementary Material); (c) lowest number of cells in phase S + G2/M (Fig. S6, Supplementary Material) as compared to positive control (PHA stimulated cells). The results related to BrdU incorporation are in agreement with our previous observation which showed also suppression in lymphocyte blast transformation test induced by compound **7**/PHA (Fig. S5) and compound **7**/anti-CD3 antibodies (Fig. 3). γH2AX is a specific cellular indicator of double-stranded DNA break during the biological process (for example meiosis, cell cycle, aging) and during exposure to harmful physical and chemical agents (for example, UV, ROS, lack of oxygen). Some constitutive level of γH2AX exists that is dependent on the cell type and the phase of the cell cycle. Here, we considered this factor as an indicator of replicating DNA during the cell cycle [25, 26]. The mitogen activator such as PHA strongly induced metabolic reaction and reactive oxygen spieces production. In our experiment, these processes were blocked by selected hydrazide **7**. It is important to note that the redox status is higher during inflammation, so the results could be evidence that the compound **7** relieves inflammation [27]. Other experiments have shown that derivate **7** also stopped the DNA synthesis machinery on phase G1 (Fig. S6). The fraction of cells in the S + G2/M phase of cell cycle in compound **7**-treated lymphocytes stimulated with PHA was lower than that in lymphocytes stimulated with PHA only. The results suggest immunosuppressive activities of this derivate.

### Antibacterial activity of **5**–**8**

Compounds **5**–**8** were evaluated for their antibacterial activity (Table S6, Supplementary Material). The tested compounds were more effective against Gram-positive than Gram-negative bacteria. However, the obtained MIC values ≥ 100 µg/cm^3^ revealed that they were devoid of significant antibacterial activity. The obtained MIC values were also lower than those reported for 1,2,4-triazole derivatives [8] obtained by cyclization of compounds **5**–**8**.

## Conclusions

A series of new hydrazides were synthesized in the reaction of N^3^-substituted amidrazones with cyclic anhydrides and their biological activities were experimentally evaluated. The studies revealed that derivatives **6**–**8** possess antiproliferative properties. Among them, compound **7** seems to have the strongest anti-inflammatory potential. We observed that this compound was able to inhibit lymphocyte proliferation in response to both polyclonal activators (PHA, anti CD3 antibodies) in a dose-dependent manner. This antiproliferative effect was not due to increased cell death, but due to its ability to induce cell cycle arrest in the G1 phase. Here, we have the evidence that **7** is non-toxic and inhibits lymphocyte activation. These properties indicate that it could be potentially useful as an anti-inflammatory agent.

## Experimental

The reagents were purchased from Sigma-Aldrich Chemicals (St. Louis, MO, USA). All reactions were controlled by reversed-phased TLC chromatography (HPTLC RP-18W nano-silica gel aluminum plates (60 Å medium pore diameter, 0.150 mm-thick layer, Fluka, Germany) using methanol–water mixture (1:1) as a mobile phase. Elemental analyses (C, H, N) were performed using a CHN Perkin-Elmer 2400 instrument. Melting points were measured on a MEL-TEMP apparatus. IR spectra were recorded with a Shimadzu FTIR 8400S spectrometer in KBr pallets. ^1^H and ^13^C NMR (including DEPT 90° and 135°) spectra were measured by a Bruker Avance III 400 MHz NMR spectrometer, at 300 K in DMSO-*d*_6_. The ^1^H and ^13^C chemical shifts were referenced to TMS using residual ^1^H and ^13^C DMSO-*d*_5_ solvent signals as primary references (adjusted at 2.50 and 40.0 ppm, respectively). Additionally, ^1^H-^13^C two-dimensional HMQC- and HMBC-NMR spectra were recorded under the following parameters: ^1^*J*_H–C_ = 145 Hz and ^n^*J*_H–C_ = 7.5 Hz; π/2 pulse lengths: 9.5 μs for ^1^H and 13.1 μs for ^13^C; acquisition time: 0.15 s for ^1^H-^13^C HMQC and 0.2 s for ^1^H-^13^C HMBC; relaxation delay 1.5 s.

### General method for the preparation of compounds **5**–**8**

In each case, a mixture of amidrazone **1**–**4** (1 mmol) [20] and itaconic anhydride (1 mmol) was dissolved in 30 cm^3^ anhydrous diethyl ether and stirred for 2 h at ambient temperature. The obtained precipitates of **5**–**8** were collected by filtration and washed with anhydrous diethyl ether. Compounds **5** and **6** were additionally purified by crystallization from ethanol and ethanol–water mixture (1:1), respectively.

In the spectroscopic characterization of **5**–**8** described below, the ^1^H and ^13^C NMR chemical shifts are presented as unassigned, only with distinguishing C, CH, CH_2_, and CH_3_ carbons by C, C^H^, C^2H^, and C^3H^ symbols (as concluded from ^13^C DEPT). Symbol * denotes that the two listed ^1^H or ^13^C signals derive from **A** (major) and **B** (minor) tautomers of **5**–**8**, their *δ*^1^H or *δ*^13^C parameters being listed in the “**A** and **B**” order (as revealed by ^1^H integration and ^1^H-^13^C HMQC or HMBC spectra). The full ^1^H and ^13^C assignments have been discussed in the Supplementary Material and presented in Tables S1–S3.

#### 2-Methylidene-4-oxo-4-[2-[pyridin-2-yl(pyridin-2-ylamino)methylidene]hydrazinyl]butanoic acid (**5**, C_16_H_15_N_5_O_3_)

Yield 70%; m.p.: 147–148 °C; ^1^H NMR (DMSO-*d*_6_): *δ* = 3.71 and 3.28 (2H)*, 5.74 and 5.76 (1H)*, 6.17 and 6.16 (1H)*, 6.85 (1H), 6.96 (1H), 7.41 (1H), 7.62 (1H), 7.88 (1H), 8.02 (2H), 8.49 (1H), 9.23 (1H, broad, ν_1/2_ = ca. 30 Hz), 10.91 and 11.36 (1H)*, ca. 12.5 (1H, broad, ca. 120 Hz) ppm; ^13^C NMR (DMSO-*d*_6_): *δ* = 36.4 and 38.5 (1C^2H^)*, 112.7 (1C^H^), 116.6 (1C^H^), 122.5 (1C^H^), 124.6 (1C^H^), 127.5 and 128.2 (1C^2H^)*, 136.5 and 136.0 (1C)*, 137.5 (1C^H^), 138.6 (1C^H^), 138.9 and 141.7 (1C)*, 147.4 (1C^H^), 148.5 (1C^H^), 152.6 (1C), 154.5 (1C), 172.1 and 166.2 (1C)*, 168.2 and 168.0 (1C)* ppm; IR (KBr): $\bar{\nu }$ = 3439, 3211, 3067, 1702, 1604, 1526, 1478 cm^−1^; *R*_*f*_ = 0.39.

#### 2-Methylidene-4-[2-[[(4-nitrophenyl)amino](pyridin-2-yl)methylidene]hydrazinyl]-4-oxobutanoic acid (**6**, C_17_H_15_N_5_O_5_)

Yield 80%; m.p.: 139–142 °C; ^1^H NMR (DMSO-*d*_6_): *δ* = 3.73 and 3.27 (2H)*, 5.74 and 5.72 (1H)*, 6.18 and 6.14 (1H)*, 6.67 (2H), 7.45 (1H), 7.92 (1H), 8.02 (3H), 8.51 (1H), 9.31 and 9.41 (1H)*, 10.45 and 10.60 (1H)*, ca. 12.5 (1H, broad, ca. 120 Hz) ppm; ^13^C NMR (DMSO-*d*_6_): *δ* = 36.6 and 37.9 (1C^2H^)*, 116.9 (2C^H^), 123.0 (1C^H^), 125.2 (1C^H^), 125.4 (2C^H^), 127.7 and 128.0 (1C^2H^)*, 136.4 and 136.0 (1C)*, 137.7 (1C^H^), 139.4 and 142.6 (1C)*, 139.9 (1C), 149.0 (1C), 149.2 (1C^H^), 151.8 (1C), 172.5 and 167.0 (1C)*, 168.1 and 168.1 (1C)* ppm; IR (KBr): $\bar{\nu }$ = 3414, 3194, 3080, 2988, 1709, 1670, 1593, 1551, 1526, 1327 cm^−1^; *R*_*f*_ = 0.45.

#### 2-Methylidene-4-[2-[[(4-methylphenyl)amino](pyridin-2-yl)methylidene]hydrazinyl]-4-oxobutanoic acid (**7**, C_18_H_18_N_4_O_3_)

Yield 71%; m.p.: 74–78 °C; ^1^H NMR (DMSO-*d*_6_): *δ* = 2.36 (3H), 3.54 (2H), 5.58 (1H), 6.14 (1H), 7.19 (2H), 7.27 (2H), 7.35 (1H), 7.89 (1H), 7.94 (1H), 8.33 (1H), 8.46 and 8.51 (1H)*, 9.77 and 10.01 (1H)*, ca. 12.5 (1H, broad, ν_1/2_ = ca. 200 Hz) ppm; ^13^C NMR (DMSO-*d*_6_): *δ* = 21.2 (C^3H^), 27.9 (1C^2H^), 124.2 (1CH), 124.6 (1CH), 127.4 (1C^2H^), 127.6 (2CH), 130.2 (2CH), 132.8 (1C), 136.4 (1C), 137.5 (1CH), 139.0 (1C), 141.7 (1C), 147.3 (1C), 149.5 (1CH), 167.5 (1C), 168.2 and 171.9 (1C)* ppm; IR (KBr): $\bar{\nu }$ = 3431, 3215, 3096, 2922, 1707, 1514, 1460 cm^−1^; *R*_*f*_ = 0.43.

#### 2-Methylidene-4-[2-[[(4-nitrophenyl)amino](phenyl)methylidene]hydrazinyl]-4-oxobutanoic acid (8, C_18_H_16_N_4_O_5_)

Yield 91%; m.p.: 128–132 °C; ^1^H NMR (DMSO-*d*_6_): *δ* = 3.70 and 3.27 (2H)*, 5.72 and 5.71 (1H)*, 6.16 and 6.13 (1H)*, 6.67 (2H), 7.41 (1H), 7.43 (2H), 7.61 (2H), 8.05 (2H), 9.24 and 9.33 (1H)*, 10.48 and 10.66 (1H)*, ca. 12.5 (1H, broad, ca. 200 Hz) ppm; ^13^C NMR (DMSO-*d*_6_): *δ* = 36.6 and 37.9 (1C^2H^)*, 116.5 (2C^H^), 125.7 (2C^H^), 127.6 and 127.6 (1C^2H^)*, 127.9 (2C^H^), 129.1 (2C^H^), 130.5 (1C^H^), 133.9 (1C), 136.5 and 136.2 (1C)*, 139.6 and 143.2 (1C)*, 139.7 (1C), 149.2 (1C), 172.4 and 166.8 (1C)*, 168.1 and 168.1 (1C)* ppm; IR (KBr): $\bar{\nu }$ = 3436, 3284, 3160, 2963, 1697, 1663, 1593, 1553, 1522, 1331 cm^−1^; *R*_*f*_ = 0.33.

### Single-crystal X-ray diffraction analysis

Crystal data: (**5**) C_16_H_15_O_3_N_5_, *M*_w_= 325.33 g mol^−1^, monoclinic, space group *P*2_1_/c, *a *= 9.507(1) Å, *b *= 13.402(2) Å, *c *= 12.785(2) Å, * β*= 109.69(1)°, *V *= 1533.6(4) Å^3^, *Z *= 4, *d*_calc_= 1.409 g cm^−3^, *μ *= 0.101 mm^−1^, data/restraints/parameters 3523/0/237, *R*_int_ = 0.025, *R*_1_ = 0.038, *wR*_2_(all refl.) = 0.100, GooF = 1.08; Δ*ρ*_max_, Δ*ρ*_min_: 0.46 and − 0.18 e Å^−3^.

Single crystals of **5**, suitable for X-ray diffraction studies, were grown by crystallization from ethanol. The crystallographic measurements were performed on an Oxford Diffraction Xcalibur CCD diffractometer with graphite-monochromatized Mo K_α_ radiation (*λ* = 0.7107 Å). The data were collected at 100(2) K using the *ω* scan technique with an angular scan width of 1.0°. The CRYSALIS set of programs [28] was used for data collection, cell refinement and data reduction. A multi-scan absorption correction was applied. The structure was solved by the direct methods using SHELXS-97 [29] and refined by the full-matrix least squares on *F*^2^ using SHELXL-97 [29]. All non-H atoms were refined with the anisotropic displacement parameters. The carboxylic, amine, amide, and methylene H atoms were found in the difference-Fourier maps and refined with the isotropic displacement parameters. All remaining ones were placed in the geometrically calculated positions and refined using the riding model with *U*_iso_(H) = 1.2*U*_eq_(C).

CCDC-1537303 contains the supplementary crystallographic data for this paper. These data can be obtained free of charge from the Cambridge Crystallographic Data Centre via www.ccdc.cam.ac.uk/data_request/cif.

### Biological assays in vitro

Human peripheral blood mononuclear cells (PBMC) were isolated from buffy coats obtained from normal blood donors of median age equaling 30 years old (range 20–35) by density gradient centrifugation (LSM 1077, PAA). For all experiments, freshly isolated PBMC were used. PBMC (2 × 10^6^ cells/cm^3^) were subjected to culture with studied compounds in RPMI 1640 medium (Cytogen) supplemented with 5% heat-inactivated human serum (AB Rh +). The compounds **5**–**8** and ibuprofen (IBU) were initially dissolved in DMSO (Sigma), then in culture medium to obtain concentrations 1, 10, and 50 μg/cm^3^.

### Cell toxicity analyses

PBMC and compounds **5**–**8** were incubated alone in 24-well polypropylene, non-adherent plate (Cytogen) for 24 h. Control cultures contained DMSO or ibuprofen (IBU). After stimulation, apoptosis was assessed by annexin V–FITC and propidium iodide (FITC Annexin V Apoptosis Detection Kit I, Becton–Dickinson Pharmingen). Then, cells were analyzed in FACScan flow cytometer (Becton–Dickinson). Flow cytometry acquisition and analysis were performed on at least 10,000 acquired events. Cytometric data were analyzed using FlowJo version 7.6.1 software (Tree Star) [13].

### Lymphocyte proliferation assay

PBMC (180 mm^3^, 2 × 10^6^ cells/cm^3^) and 10 mm^3^ of culture medium (control) or compounds **5**–**8** (1, 10, and 50 μg/cm^3^) and anti-CD3 antibody (4 μg/cm^3^, IgG1, Immunotech) or PHA (0.5 μg/cm^3^, Sigma) were cultured for 72 h in a flat-bottom 96-well plate (Becton-Dickinson). Control cultures contained DMSO (the highest dose of DMSO used as a solvent for compounds) or IBU incubated with anti-CD3 or PHA alone. Lymphocyte proliferation was assessed by pulsing the cells with 5 μCi ^3^H thymidine (Amersham) for the last 18 h of the incubation period. The cultures were then harvested onto glass filter strips using the automated multisample harvester (Skatron) and analyzed for ^3^H thymidine incorporation by liquid scintillation counting—Betamic V (Kontron Instruments, USA) [13]. Statistical analysis was conducted with Statistica 12.5 software (StatSoft). The normal distribution was checked using the Shapiro–Wilk test. The data set was found to be abnormally distributed so the results were compared using the Mann–Whitney’s* U*-test. Statistical significance was considered at *p* < 0.05.

### Flow cytometric detection of BrdU-incorporated cells, expression of γH2A, cleaved PARP, and total DNA

PBMC were stimulated with compound 7 (1, 10, and 50 μg/cm^3^) and/or PHA (0.5 μg/cm^3^, Sigma) for 72 h in Falcon round-bottom polypropylene tubes (Becton-Dickinson), then labeled (1 h) with 50 μM of 5′-bromo-2′-deoxyuridine (BrdU). Then, BrdU-pulsed cells were washed once with staining buffer (FBS), two times fixed and permeabilized with single-step fixation and permeabilization reagent, containing a mixture of the fixative paraformaldehyde and the detergent saponin (Cytofix/Cytoperm Fixation/Permeabilization solution, BD Pharmingen). To expose incorporated BrdU, the cells were treated (1 h, 37 °C) with DNase. Afterward, cells were immunofluorescent stained (20 min at room temperature) with appropriate intracellular antigen-specific antibodies: PerCP-Cy5.5 anti-BrdU, Alexa Fluor 647 Mouse anti-H2AX (pS139), and PE anti-cleaved PARP (Asp214). Cells were washed once and resuspended with 1 cm^3^ of DAPI solution (1 μg/cm^3^). Stained cells were harvested and analyzed using FACSCanto II flow cytometer (BD). Flow cytometry acquisition and analysis were performed on at least 10,000 acquired events. Cytometric data were analyzed using FlowJo version 7.6.1 software (Tree Star) [13].

### Antibacterial activity

The broth microdilution method, in 96-well microtiter plates (Kartell), was used to evaluate the antimicrobial activity of compounds **5**–**9**. The following bacterial strains were tested: Gram-negative: *Escherichia coli* ATCC 25922, *Pseudomonas aeruginosa* ATCC 27853, and *Yersinia enterocolitica* O_3_; Gram-positive: *Staphylococcus aureus* ATCC 25923, *Enterococcus faecalis* ATCC 29212, *Sarcina lutea*, *Mycobacterium smegmatis*, and *Nocardia corralina.* The tested strains at final concentration of 10^5^ CFU/cm^3^ were inoculated into a liquid Luria–Bertani (LB) medium in the presence of different concentrations (25, 50, 75, 100, and 250 μg/cm^3^) of compounds dissolved in DMSO. Tests were performed in triplicate for each concentration, in all the tests DMSO was used as the control. The microbial growth was measured at a wavelength of 550 nm after 18 h incubation. The MIC (minimum inhibitory concentration) values were defined as the  lowest concentration of tested compounds that inhibited microbial growth as compared to the drug-free control.

## Electronic supplementary material

Below is the link to the electronic supplementary material.

Additional spectral and crystallographic data, cytograms, MIC values, are available as supplementary material. (DOC 733 kb)

## References

[1] Siódmiak T, Ziegler-Borowska M, Marszałł MP (2013). J Mol Catal B Enzym.

[2] Shiau L-D, Liu K-F, Hsu Y-C (2017). Chem Eng Res Des.

[3] Obad J, Šušković J, Kos B (2015). Eur J Pharm Sci.

[4] Khodov IA, Efimov SV, Klochkov VV, Alper GA, Batista de Carvalho LA (2014). Eur J Pharm Sci.

[5] Trelle S, Reichenbach S, Wandel S, Hildebrand P, Tschannen B, Villiger PM, Egger M, Juni P (2011). Br Med J.

[6] Modzelewska-Banachiewicz B, Ucherek M, Zimecki M, Kutkowska J, Kaminska T, Morak-Młodawska B, Paprocka R, Szulc M, Lewandowski G, Marciniak J, Bobkiewicz-Kozlowska T (2012). Arch Pharm (Weinheim).

[7] Kozminykh VO (2006). Pharm Chem J.

[8] Paprocka R, Modzelewska-Banachiewicz B, Kutkowska J, Pawłowski K, Piątkowska-Chmiel I, Jagiełło-Wójtowicz E (2017). Acta Pol Pharm.

[9] Abdaleh MA (2016). Asian J Chem.

[10] Abdaleh MA, El-Abadelah MM, Sabri SS, Mohammed HH, Zihlif MA, Voelter W (2014). Z Naturforsch B Chem Sci.

[11] Modzelewska-Banachiewicz B, Banachiewicz JJ, Chodkowska A, Jagiello-Wojtowicz E, Mazur L (2004). Eur J Med Chem.

[12] Aly AA, Nour-El-Din AM (2008) Arkivoc (i):153

[13] Paprocka R, Wiese M, Eljaszewicz A, Helmin-Basa A, Gzella A, Modzelewska-Banachiewicz B, Michalkiewicz J (2015). Bioorg Med Chem Lett.

[14] Paprocka R, Modzelewska-Banachiewicz B, Wiese M, Eljaszewicz A, Michalkiewicz J (2012). Acta Pol Pharm.

[15] Refat HM, Fadda AA (2013). Eur J Med Chem.

[16] Matei L, Bleotu C, Baciu I, Diaconu CC, Hanganu A, Banu O, Ionita P, Paun A, Tatibouët A, Zarafu I (2015). Bioorg Med Chem.

[17] Malhotra M, Sharma R, Rathee D, Phogat P, Deep A (2014). Arabian J Chem.

[18] Theuretzbacher U (2013). J Glob Antimicrob Resist.

[19] Morjan RY, Mkadmh AM, Beadham I, Elmanama AA, Mattar MR, Raftery J, Pritchard RG, Awadallah AM, Gardiner JM (2014). Bioorg Med Chem Lett.

[20] Modzelewska B, Pyra E (1995–1996) Annales UMCS sec. AA L/LI9, 50/51:111

[21] Ianelli S, Pelosi G, Ponticelli G, Cocco MT, Onnis V (2001). J Chem Crystallogr.

[22] Razzaq TM, Ozegbe P, Jury EC, Sembi P, Blackwell NM, Kabouridis PS (2004). Immunology.

[23] Cheng J, Montecalvo A, Kane LP (2011). Immunol Res.

[24] Wimer BM (1996). Cancer Biother Radiopharm.

[25] Liu Y-P, Chen H-L, Tzeng C-C, Lu P-J, Lo C-W, Lee Y-C, Tseng C-H, Chen Y-L, Yan C-N (2013). Breast Cancer Res Treat.

[26] Tanaka T, Kajstura M, Halicka HD, Traganos F, Darzynkiewicz Z (2007). Cell Prolif.

[27] Checker R, Sharma D, Sandur SK, Subrahmanyam G, Krishnan S, Poduval TB, Sainis KB (2010). J Cell Biochem.

[28] Agilent Technologies (2013) Crysalis Pro. Yarnton, Oxfordshire, England, UK

[29] Sheldrick GM (2008). Acta Cryst.

