# Supplementary material for: Synthesis and evaluation of new amidrazone-derived hydrazides as a potential anti-inflammatory agents
Source: Monatsh Chem. 2018 Jun 27;149(8):1493–500. doi: 10.1007/s00706-018-2197-8 (PMC6060958; doi:10.1007/s00706-018-2197-8)
Supplement: Supplementary file 1 — Additional spectral and crystallographic data, cytograms, MIC values, are available as supplementary material. (DOC 733 kb) [file 706_2018_2197_MOESM1_ESM.doc]

**Supporting information for publication**

### Synthesis and Evaluation of New Amidrazone-Derived Hydrazides as a Potential Anti-inflammatory Agents

**Renata Paprocka^1^ ● Małgorzata Wiese^2^ ● Anna Helmin-Basa^2^ ● Mazur Liliana^3^ ● Jolanta Kutkowska^4^ ● Jacek Michałkiewicz^2,5^ ● Bożena Modzelewska-Banachiewicz^1^ ● Leszek Pazderski^6^**

____

🖂 Renata Paprocka,

[renata.bursa@cm.umk.pl](mailto:renata.bursa@cm.umk.pl)

^1^ Department of Organic Chemistry, Faculty of Pharmacy, Nicolaus Copernicus University in Toruń, Bydgoszcz, Poland

^2^ Department of Genetics and Microbiology, Maria Curie-Sklodowska University, Lublin, Poland

^3^ Faculty of Chemistry, Maria Curie-Skłodowska University, Lublin, Poland

^4^ Department of Immunology, Faculty of Pharmacy, Nicolaus Copernicus University in Toruń, Bydgoszcz, Poland

^5^ Department of Clinical Microbiology and Immunology, The Children's Memorial Health Institute, Warsaw, Poland

^6^ Department of Analytical Chemistry and Applied Spectroscopy, Faculty of Chemistry, Nicolaus Copernicus University in Toruń, Toruń, Poland

The assignment of ^1^H and ^13^C resonances has been based on the analysis of the ^1^H-^13^C HMQC and ^1^H-^13^C HMBC spectra. Generally, for all **5-8** molecules three main moieties, in which the long-range correlations are detected during the ^1^H-^13^C HMBC experiments, can be distinguished: (a) aliphatic chain from the –COOH group to the –NH– or –N= nitrogen (in the amide-hydrazone **A** or hydrazide imide **B** tautomers, respectively); (b) 2-pyridyl (**5**-**7**) or phenyl (**8**) aromatic ring bound to the carbon atom of the R^2^–NH–C(R^1^)= or R^2^–N=C(R^1^)– fragment, *i.e.* R^1^; (c) 2-pyridyl (**5**), 4-nitrophenyl (**6**, **8**) or 4-methylphenyl (**7**) aromatic ring bound to the nitrogen atom of the R^2^–NH–C(R^1^)= or R^2^–N=C(R^1^)– fragment, *i.e.* R^2^.

Within the (a) aliphatic chain, the most characteristic are the following long-range (over two or three bonds) ^1^H-^13^C correlations: between both vinyl =CH_2_ hydrogens (inequivalent due to their different geometric positions in respect to the other parts of the molecule) and the quaternary >C= atom bonded to COOH, the carboxylic –COOH carbon and the methylene –CH_2_– carbon, as well as between both methylene –CH_2_– hydrogens (equivalent due to the rotation around the neighbouring C–C single bonds) and the carbonyl >C=O carbon, the already mentioned quaternary >C= atom, the carboxylic –COOH carbon and the vinyl =CH_2_ carbon. Moreover, the –NH–CO– hydrogen correlates with the carbonyl >C=O carbon, the methylene –CH_2_– carbon and the R^1^-bonded quaternary >C= atom. To make this issue more clear, the ^1^H-^13^C long-range correlation pattern is summarized in Table S1.

Generally, the ^1^H-^13^C correlations in the (a) aliphatic chain as well as the respective distinct ^1^H and ^13^C signals are observed separately for **A** and **B** tautomers (except for the carboxylic –COOH proton, which significantly broadened ^1^H peaks are shared by both forms, probably due to its mobility). However, in case of **7** some noticeable differences between the δ^1H^ and δ^13C^ chemical shifts for **A** and **B** occur only for the hydrogens and carbons in the hydrazone amide =N–NH–CO– or hydrazide –NH–NH–CO– group, being undetectable for the remaining part of the (a) aliphatic chain.

The short-range (over one bond) correlations, revealed by ^1^H-^13^C HMQC experiments, are observed within the (a) aliphatic chain for the vinyl =CH_2_ group and the methylene –CH_2_– group, again separately for **A** and **B**.

Both long- and short-range ^1^H-^13^C correlations, observed inside the R^1^ or R^2^ aromatic rings, are typical for the concerned pyridyl or phenyl substituents (including those involving the methyl –CH_3_ side group in **7**) and there is no need to discuss them in a detailed way.

The assigned ^1^H and ^13^C chemical shifts (δ^1H^, δ^13C^) for **5-8** are listed in Tables S2 and S3.

**Table S1** ^1^H-^13^C two-bond (+) and three-bond (*) correlations observed in the ^1^H-^13^C HMBC spectra of **5**-**8**, within the (a) aliphatic chain moiety (X denotes the same position of H and C atoms – one-bond correlations detected in the ^1^H-^13^C HMQC spectra)

| Hydrogen(s)  -------------------------  Carbon | =CH_2_ | –CH_2_– | –NH–CO– |
| --- | --- | --- | --- |
| >C= (bonded to COOH) | + | + |  |
| –COOH | * | * |  |
| –CH_2_– | * | X | * |
| >C=O |  | + | + |
| >C= (bonded to R^1^) |  |  | * |
| =CH_2_ | X | * |  |

**Table S2** ^1^H NMR chemical shifts for **5-8** (in DMSO-d_6_)

Aliphatic chain (a)

| Compound (**A** and **B**  tautomers) | –COOH | –CH_2_– | –NH–CO– | –NH– | =CH_2_  (H^I^, H^II^) |
| --- | --- | --- | --- | --- | --- |
| **5** | **A+B**  *ca.* 12.5 | **A** 3.71  **B** 3.28 | **A** 10.91  **B** 11.36 | **A+B**  9.23 | **A** 6.17, 5.74  **B** 6.16, 5.76 |
| **6** | **A+B**  *ca.* 12.5 | **A** 3.73  **B** 3.27 | **A** 10.45  **B** 10.60 | **A** 9.31  **B** 9.41 | **A** 6.18, 5.74  **B** 6.14, 5.72 |
| **7** | **A+B**  *ca.* 12.5 | **A+B**  3.54 | **A** 9.77  **B** 10.01 | **A** 8.46  **B** 8.51 | **A+B**  6.14, 5.58 |
| **8** | **A+B**  *ca.* 12.5 | **A** 3.70  **B** 3.27 | **A** 10.48  **B** 10.66 | **A** 9.24  **B** 9.33 | **A** 6.16, 5.72  **B** 6.13, 5.71 |

*Aromatic ring R^1^* – 2-pyridyl

| Compound | H3’ | H4’ | H5’ | H6’ |  |
| --- | --- | --- | --- | --- | --- |
| **5** | 7.41 | 7.88 | 8.02 | 8.49 |  |
| **6** | 7.45 | 7.92 | 8.02 | 8.51 |  |
| **7** | 7.35 | 7.89 | 7.94 | 8.33 |  |

*Aromatic ring R^1^* – phenyl

| Compound | H2’ | H3’ | H4’ |
| --- | --- | --- | --- |
| **8** | 7.61 | 7.43 | 7.41 |

*Aromatic ring R^2^* – 2-pyridyl

| Compound | H3’’ | H4’’ | H5’’ | H6’’ |
| --- | --- | --- | --- | --- |
| **5** | 6.85 | 7.62 | 6.96 | 8.02 |

*Aromatic ring R^2^* – 4-nitrophenyl or 4-methylphenyl

| Compound | H2’’ | H3’’ | CH_3_ |
| --- | --- | --- | --- |
| **6** | 6.67 | 8.02 | none |
| **7** | 7.19 | 7.27 | 2.36 |
| **8** | 6.67 | 8.05 | none |

**Table S3** ^13^C NMR chemical shifts for **5-8** (in DMSO-d_6_)

Aliphatic chain (a)

| Compound, (**A** and **B** tautomers) | –COOH | >C=  bonded to COOH | –CH_2_– | –NH–CO– | >C=  bonded to R^1^ | =CH_2_ |
| --- | --- | --- | --- | --- | --- | --- |
| **5** | **A** 168.2  **B** 168.0 | **A** 136.5  **B** 136.0 | **A** 36.4 **B** 38.5 | **A** 172.1  **B** 166.2 | **A** 138.9  **B** 141.7 | **A** 127.5  **B** 128.2 |
| **6** | **A** 168.1^a^  **B** 168.1^a^ | **A** 136.4  **B** 136.0 | **A** 36.6 **B** 37.9 | **A** 172.5  **B** 167.0 | **A** 139.4  **B** 142.6 | **A** 127.7  **B** 128.0 |
| **7** | **A+B**  167.5 | **A+B**  136.4 | **A+B**  27.9 | **A** 171.9  **B** 168.2 | **A+B**  141.7 | **A+B**  127.4 |
| **8** | **A** 168.1^a^  **B** 168.1^a^ | **A** 136.5  **B** 136.2 | **A** 36.6 **B** 37.9 | **A** 172.4  **B** 166.8 | **A** 139.6  **B** 143.2 | **A** 127.6^a^  **B** 127.6^a^ |

^a^ *ca.* 0.01-0.02 ppm differences observed

*Aromatic ring R^1^* – 2-pyridyl

| Compound | C2’ | C3’ | C4’ | C5’ | C6’ |
| --- | --- | --- | --- | --- | --- |
| **5** | 152.6 | 124.6 | 137.5 | 122.5 | 148.5 |
| **6** | 151.8 | 125.2 | 137.7 | 123.0 | 149.2 |
| **7** | 147.3 | 124.6 | 137.5 | 124.2 | 149.5 |

*Aromatic ring R^1^* – phenyl

| Compound | C1’ | C2’ | C3’ | C4’ |
| --- | --- | --- | --- | --- |
| **8** | 133.9 | 127.9 | 129.1 | 130.5 |

***Aromatic ring R^2^ – 2-pyridyl***

| Compound | C2’’ | C3’’ | C4’’ | C5’’ | C6’’ |
| --- | --- | --- | --- | --- | --- |
| **5** | 154.5 | 116.6 | 138.6 | 112.7 | 147.4 |

*Aromatic ring R^2^* – 4-nitrophenyl or 4-methylphenyl

| Compound | C1’’ | C2’’ | C3’’ | C4’’ | CH_3_ |
| --- | --- | --- | --- | --- | --- |
| **6** | 149.0 | 116.9 | 125.4 | 139.9 | none |
| **7** | 139.0 | 127.6 | 130.2 | 132.8 | 21.2 |
| **8** | 149.2 | 116.5 | 125.7 | 139.7 | none |

**Table S4**. Selected geometric parameters for **5**.

| *Bond distances* /Å | | *Torsion angles* / *˚* | |
| --- | --- | --- | --- |
| C1−O1 | 1.239(1) | O1−C1−N1−N2 | -1.7(2) |
| C1−N1 | 1.339(1) | C1−N1−N2−C2 | 170.5(1) |
| N1−N2 | 1.381(1) | N1−N2−C2−N3 | 1.1(2) |
| N2−C2 | 1.287(1) | N2−C2−N3−C3 | 18.7(2) |
| C2−N3 | 1.385(1) | C2−N3−C3−N4 | -3.3(2) |
| N3−C3 | 1.395(1) | N2−C2−C8−N5 | 176.6(1) |
| C15−O2 | 1.325(1) | N3−C2−C8−N5 | 0.4(1) |
| C15−O3 | 1.218(1) | O1−C1−C13−C14 | 149.2(1) |
| C14−C16 | 1.330(2) | O2−C19−C14−C13 | -171.2(1) |

**Table S5** Geometry of the proposed hydrogen bonds in crystal **5**

| *Interaction* | *d*_D-H_/ Å | *d*_H…A_/ Å | *d*_D…A_/ Å | *d*_D-H…A_/ ° | *Symmetry code* |
| --- | --- | --- | --- | --- | --- |
| N1−H1n…N4 | 0.90(1) | 1.88(1) | 2.721(1) | 156(1) |  |
| N3−H3n…N5 | 0.88(1) | 2.00(1) | 2.581(1) | 122(1) |  |
| O2−H2o…O1 | 0.91(2) | 1.70(2) | 2.594(2) | 168(1) | -x+2, y-1/2, -z+3/2 |
| C9−H9…O2 | 0.93 | 2.73 | 3.655(2) | 175 | 2-x, y+1/2, -z+3/2 |
| C16-H16b...O2 | 0.99(1) | 2.64(2) | 3.447(1) | 139(1) | -x+2, -y, -z+1 |
| C16-H16a...O3 | 1.00(1) | 2.45(2) | 3.427(2) | 166(1) | x, -y+1/2, z-1/2 |
| C12-H12...O1 | 0.93 | 2.52 | 3.194(2) | 130 | x-1, y, z |
| C4−H4…O3 | 0.93 | 2.55 | 3.204(2) | 128 | x-1, -y+1/2, z-1/2 |
| C5−H5…O1 | 0.93 | 2.58 | 3.473(2) | 162 | x-1, -y+1/2, z-1/2 |


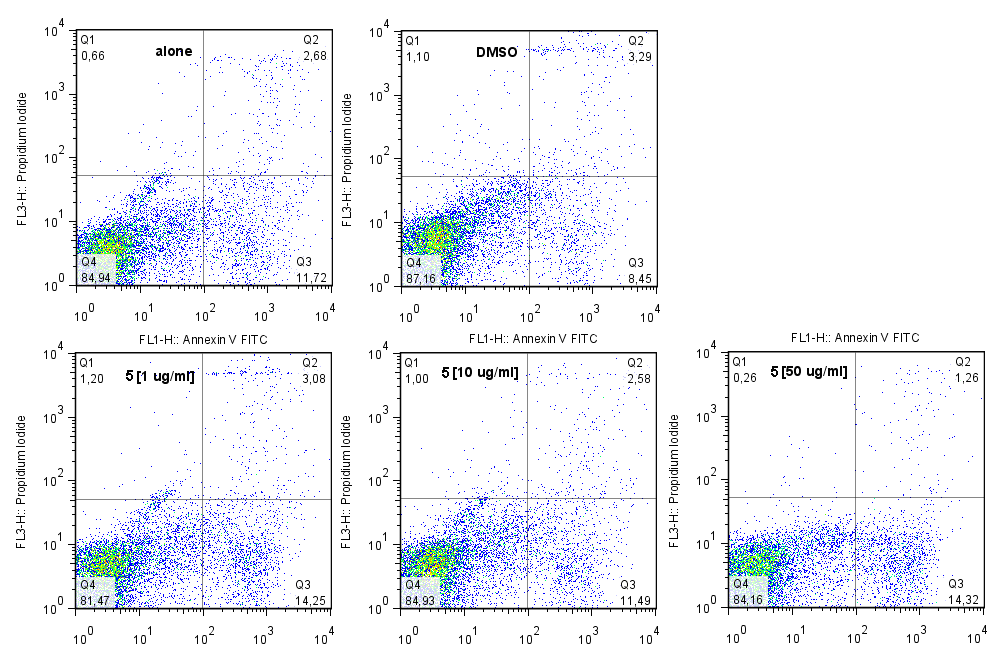


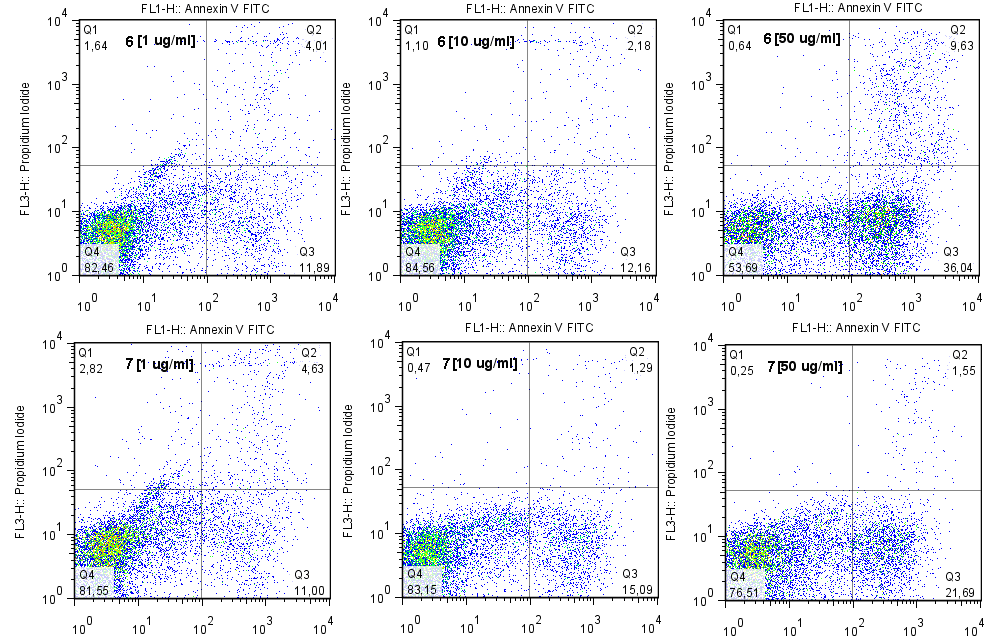


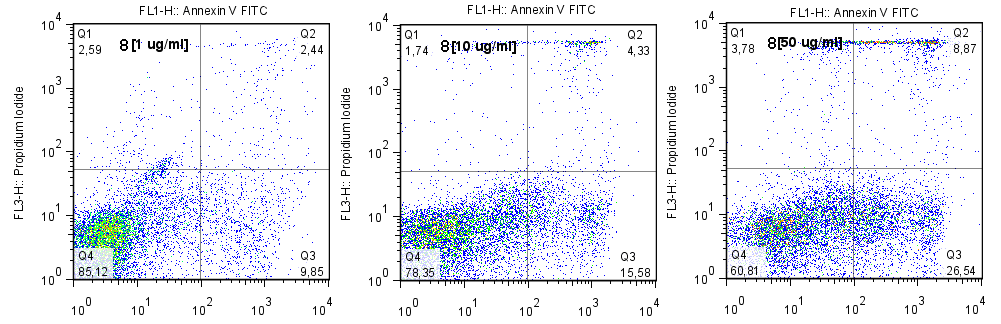


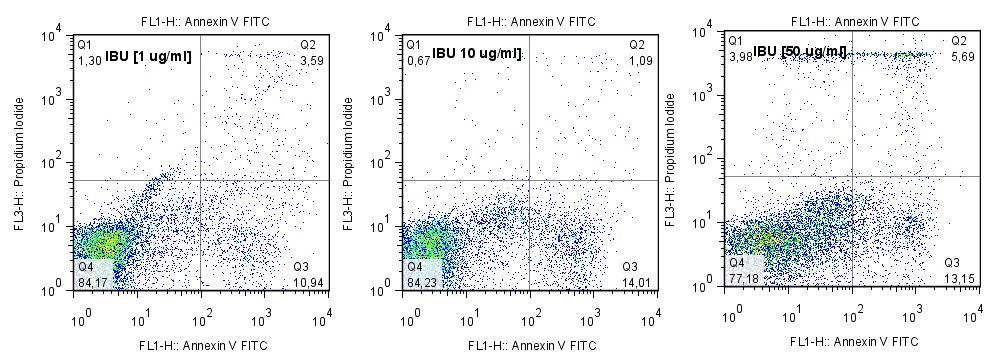


**Fig. S1**. Flow cytometry of viable and apoptotic cells in relation to control cells after 1, 10 and 50 μg/cm^3^ treatment of compounds **5-9** for 24 h. The cells are double-strained with propidium iodide and annexin V FITC. Q1: % of necrotic cells, Q2: % of late apoptotic cells, Q3: % of early apoptotic cells, Q4: % of living cells.


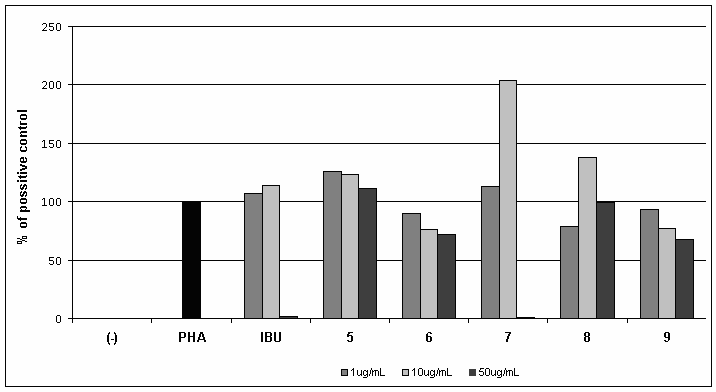
**Fig. S2.** The influence of compounds **5-9** on proliferation of human peripheral blood mononuclear cells (PBMC) induced by the PHA. Cells were treated with PHA (0.5 μg/mL) and compounds **5-9** at concentrations 1, 10 and 50 μg/mL. Ibuprofen (IBU) was used as reference drug, negative control (-) – non-stimulated PBMC. After 72 h of incubation, the proliferation of PBMC was measured using ^3^H thymidine incorporation assay. The results are shown as percentage of positive control (PHA-stimulated PBMC).


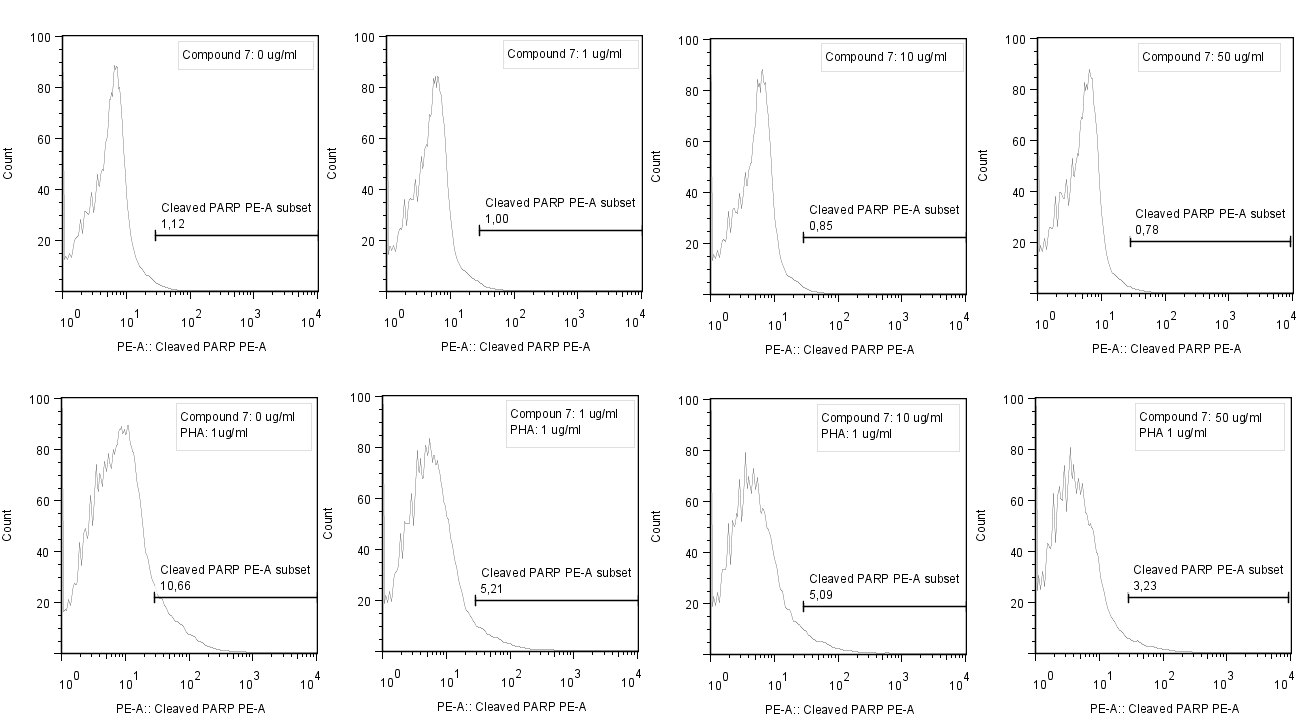


**Fig. S3.** The influence of selected compound **7** on PARP cleavage - Poly [ADP-Ribose] Polymerase - a marker of cellular apoptosis. Flow cytometric panels represent histograms with positive cells that express PARP cleavage. PARP cleavage was detected in human peripheral blood mononuclear cells culture after treated with compound **7** in three different doses (1,10, 50 ug/mL) and/or PHA for 72 h.


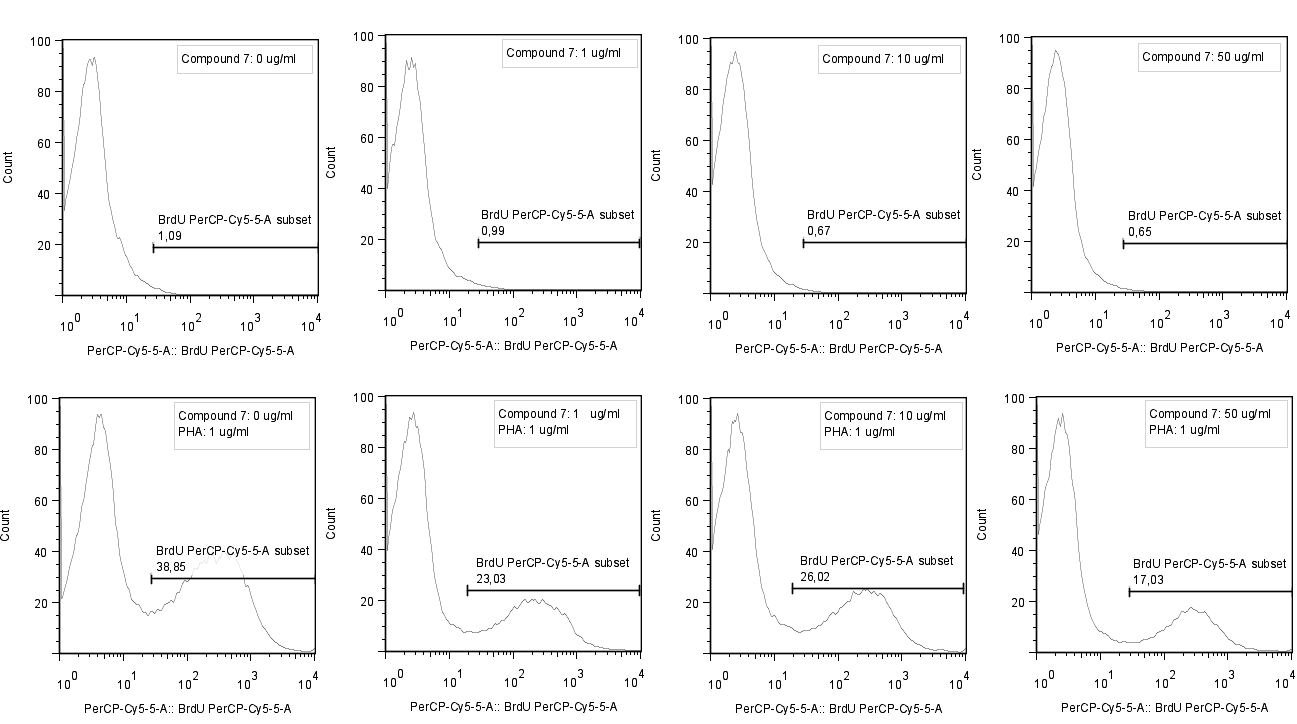


**Fig. S4.** The influence of selected compound **7** on proliferation of human peripheral blood mononuclear cells (PBMC) induced by PHA. PBMC were stimulated with compound **7** (1, 10 and 50 μg/mL) and/or PHA. Proliferative potential of cells was assessed by BrdU incorporation after 72 h and anti BrdU antibodies was used for flow cytometric detection of new synthesized cells.


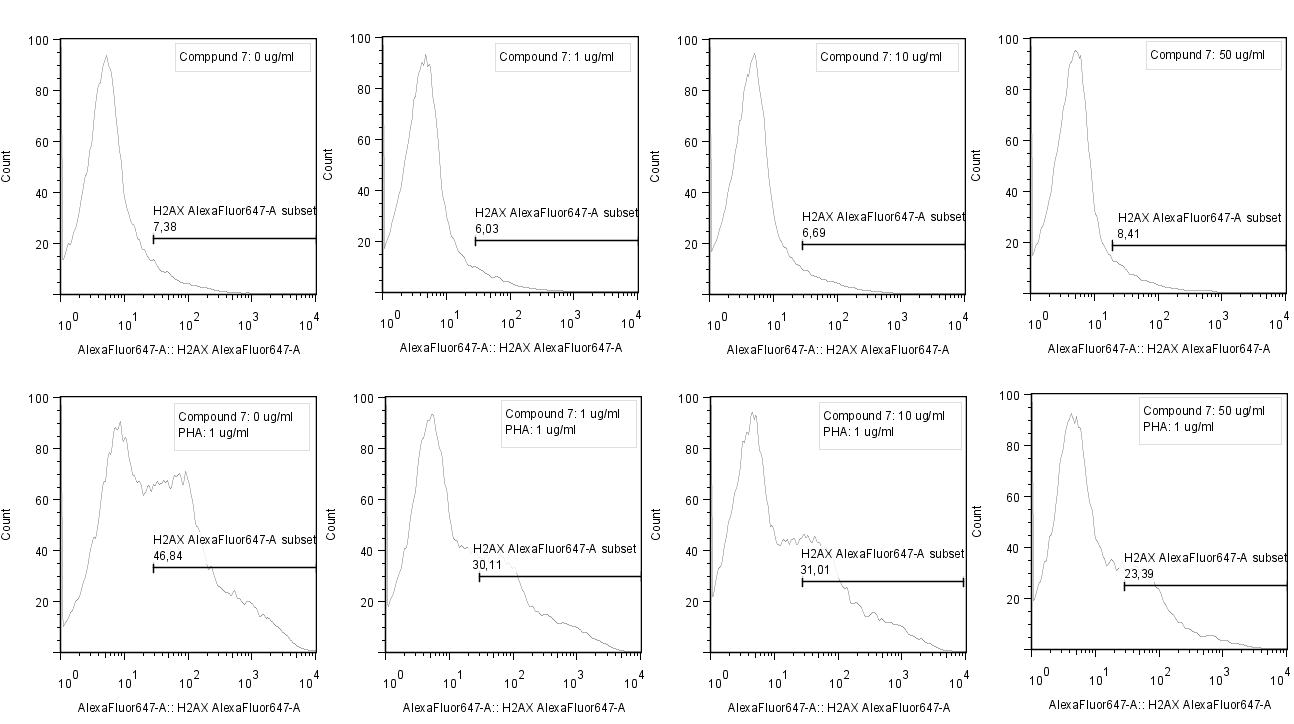


**Fig. S5.** The influence of selected compound **7** on γH2AX expression of human peripheral blood mononuclear cells (PBMC) induced by PHA. PBMC were stimulated with compound **7** (1, 10 and 50 μg/mL) and/or PHA for 72h. For flow cytometric detection of γH2AX anti- γH2AX antibodies was used.


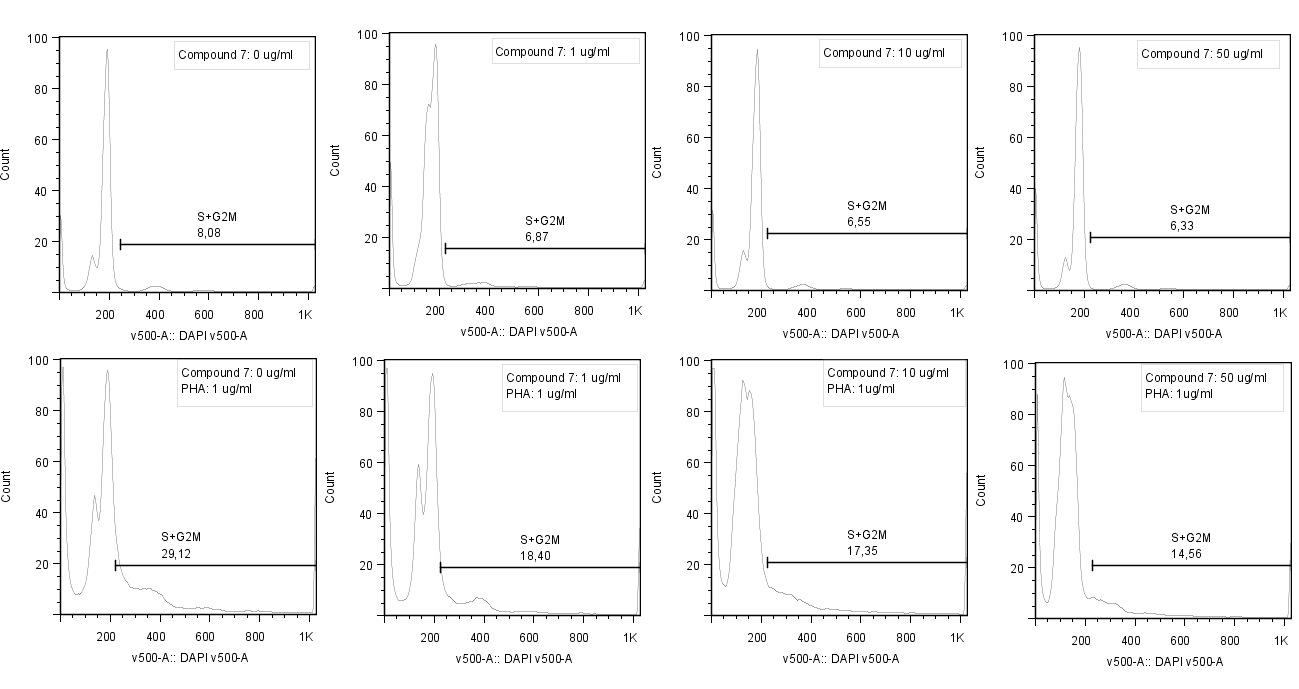


**Fig. S6.** The influence of selected compound **7** on cell cycle of human peripheral blood mononuclear cells (PBMC) induced by PHA. PBMC were stimulated with compound **7** (1, 10 and 50 μg/mL) and/or PHA for 72h. For flow cytometric detection of total DNA DAPI solution was used.

**Table S6. Minimal inhibitory concentrations of compounds 5-8**

|  | **5** | **6** | **7** | **8** |
| --- | --- | --- | --- | --- |
| *Escherichia coli* | 500 | 250 | 500 | 250 |
| *Pseudomonas aeruginosa* | 500 | 500 | 500 | 500 |
| *Yersinia enterocolitica* | 100 | 250 | 250 | 100 |
| *Staphylococcus aureus* | 250 | 100 | 100 | 100 |
| *Enterococcus faecalis* | 250 | 100 | 100 | 250 |
| *Sarcina lutea* | 250 | 250 | 100 | 250 |
| *Mycobacterium smegmatis* | 250 | 250 | 250 | 100 |
| *Nocardia corralina* | 250 | 100 | 100 | 100 |
